# Complete histologic response to chemotherapy in a patient with a mediastinal yolk sac tumor: a case report

**DOI:** 10.1186/1756-0500-7-803

**Published:** 2014-11-17

**Authors:** Yousra Akasbi, Rajae Najib, Samia Arifi, Marouane Lakranbi, Mohammed Smahi, Nawfel Mellas, Omar ELMesbahi

**Affiliations:** Medical Oncology Department Hassan II University Hospital, Fes, Morocco; Thoracic Surgery Department Hassan II University Hospital, Fes, Morocco

**Keywords:** Mediastinal nonseminomatous germ cell tumors, Complete pathologic response, Cisplatin-based chemotherapy

## Abstract

**Background:**

Malignant mediastinal germ cell tumors are a rare disease and represent only 1% to 4% of all mediastinal tumors. Gonadal germ cell tumors are generally the most common type and constitute 90% of germ cell tumors. The mediastinum is the second most frequently affected area ahead of other extragonadal areas, which include the retroperitoneum, the sacrococcygeal area, and the central nervous system. We report on the case of a mediastinal yolk sac tumor with a complete histological response to chemotherapy.

**Case presentation:**

A 26-year-old Moroccan man, without a medical or surgical history, presented with a four-month history of chest distress, dyspnea, and a frequent dry cough for the previous month. A computed tomographic scan of the chest revealed a bulky mediastinal mass, which was biopsied. Histologically, the tumoral mass proved to be a yolk sac tumor. The serum level of alpha-fetoprotein of this patient was elevated to 19052 ng/ml.

After 4 courses of preoperative chemotherapy, the patient underwent a surgical resection of the tumor, with a complete pathologic response.

At the time of writing, the patient is alive with complete remission without any evidence of recurrence.

**Conclusion:**

Primary mediastinal Yolk sac neoplasm represent a unique entity, and as such require specialized management. The diagnosis should be made not only by morphological studies but the patient’s age and the elevation of serum alpha-fetoprotein should also be considered. The utilization of cisplatin-based chemotherapy is associated with the best chance of a cure for this disease. This should be followed by surgical resection of the residual tumor in the nonseminomatous germ cell tumor.

## Background

Germ cell tumors of extragonadal origin represent only 1% to 5% of all germ cell tumors [1]. The mediastinum represents the most common site of extragonadal primaries (50% to 70%). More than half of mediastinal germ cell tumors are mature teratomas. Among malignant mediastinal germ cell tumors, 40% are seminomas and 60% are nonseminomatous tumors.

In spite of modern chemotherapy, the prognosis of mediastinal yolk sac tumors remains poor. The single most important prognostic indicator is whether the tumor mass can be completely excised before or after chemotherapy.

We report an extremely rare case of germ-cell tumor localized at the level of the anterior mediastinum, with a complete pathologic response after chemotherapy.

## Case presentation

**Figure 1:**
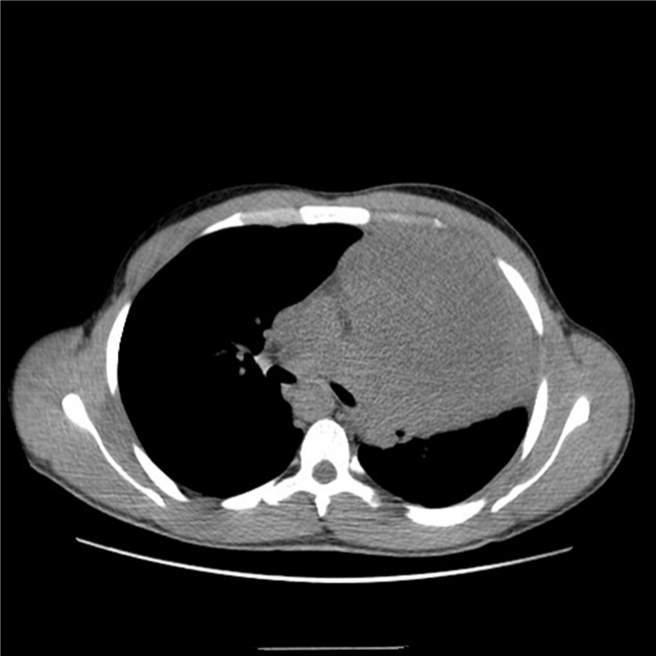
**Computed tomography scan of the chest: a huge tumor of mediastinum before treatment.**

A 26-year-old Moroccan man, without a medical or surgical history, presented with a four-month history of chest distress, dyspnea, and a frequent dry cough for the previous month. A computed tomographic scan of the chest revealed a bulky mediastinal mass, raised moderately after contrast, measuring 10 × 12cm diameter (Figure 1).

The needle core biopsy showed a malignant germ cell tumor corresponded to yolk sac tumor. The human chorionic gonadotropin levels were within normal range; but the serum level of alpha-fetoprotein of this patient was elevated to 19052 ng/ml. This supported the diagnosis of a Yolk sac tumor, a rare primary tumor within the mediastinum.

**Figure 2:**
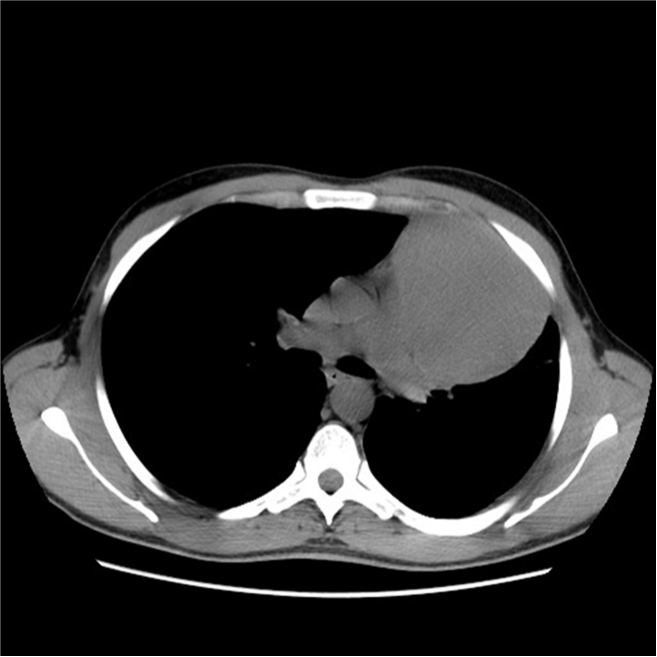
**Computed tomography scan of the chest: partial response after 4 cycles of chemotherapy.**

Preoperative chemotherapy included 4 cycles of BEP (cisplatin 20 mg/m^2^ J1-J5; bleomycin 30 mg J2, J8, J15; etoposide 100 mg/m^2^ J1-J5) was given to the patient with a partial response to chemotherapy (Figure 2). The surgery was then performed based on complete excision of all residual anterior mediastinal mass with a complete pathologic response.

**Figure 3:**
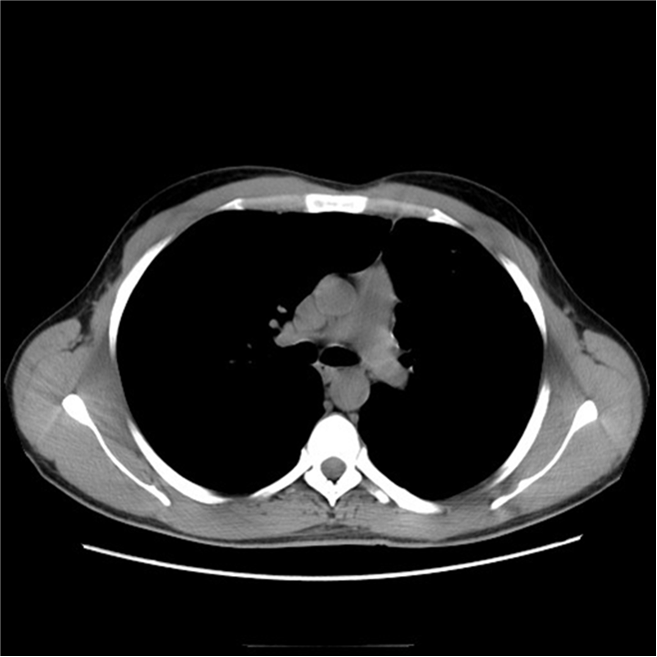
**Computed tomography scan of the chest: there is no evidence of disease 5 months after treatment.**

**Figure 4:**
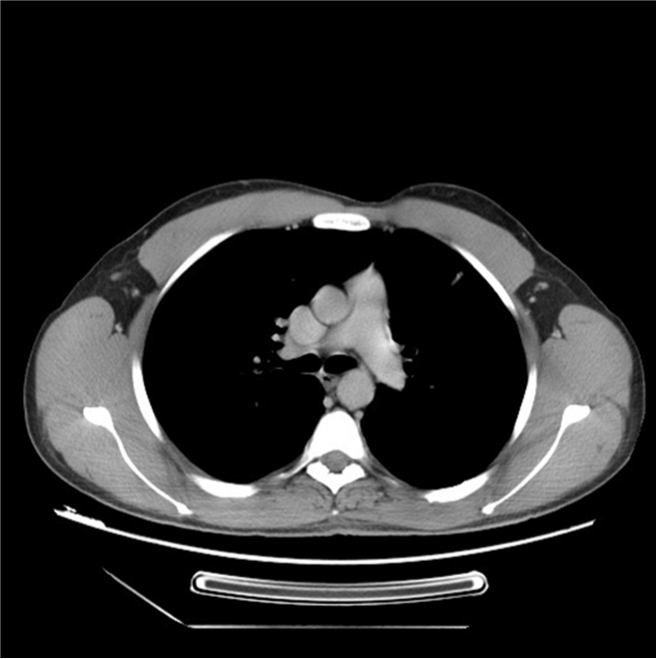
**Computed tomography scan of the chest: complete response 5 years after diagnosis.**

The patient survived 60 months after surgery with complete remission without any evidence of recurrence (Figures 3 and 4).

## Discussion

Extragonadal germ cell tumors account for 1–5% of all germ cell tumors [1]. The most widely accepted theory suggests that extragonadal germ cell tumors arise from primordial germ cells misplaced during their migration to gonads [2].

The mediastinum is the most common site of extragonadal germ cell tumors [3], constituting 50–70% of all extragonadal germ cell tumors. In adults, extragonadal germ cell tumors account for 15% of primary anterior mediastinal tumors [4].

Mediastinal nonseminomatous germ cell tumors has features that differentiate it from gonadal non seminomatous germ cell tumor, such as an association with Klinefelter syndrome and the frequent development of a hematologic malignancy [5–7].

Mediastinal nonseminomatous germ cell tumors carry a poor prognosis with 40-50% overall survival, which classified this entity as a poor prognosis group in International Germ Cell Cancer Collaborative Group (IGCCCG) [8–10].

Several causes of this unfavorable prognosis have been suggested, one of which is the bulky presentation that makes complete surgical resection impossible in patients with advanced-stages of the disease. The different histology of mediastinal non seminomatous germ cell tumors could also influence prognosis. Pure yolk sac and pure choriocarcinoma occur frequently among mediastinal nonseminomatous germ cell tumors but are rarely found among primary testicular tumors.

The current standard treatment in mediastinal non seminomatous germ cell tumors is chemotherapy combined with postchemotherapy residual mass excision, to achieve long-term survival.

Our case represent a rare case of mediastinal germ-cell tumor and show the importance of multidisciplinary approach which offer a good chance for patients to survive, we report in the Table 1 below the most important case report about mediastinal germ cell tumor.

**Table 1:** **Summary of the results of the most important case report about mediastinal germ cell tumor**

Reference	Case repot	Diagnosis	Treatment	Prognosis
**Tinica et al,** [11]	A mediastinal germ cell tumor of yolk sac type--case report.	Mediastinal Yolk sac tumor	Surgery and adjuvant chemotherapy consisting of cisplatin, vepesid and bleomycin	Poor
**Fujita et al,** [12]	Three cases of resected primary mediastinal yolk sac tumor following six courses of bleomycin, etoposide and cisplatin (BEP) combination chemotherapy	Primary mediastinal yolk sac tumor	Surgery after 6 courses of BEP chemotherapy with G-CSF support	3 years, 9 months and 5 months after operation.
**Tabuchi et al,** [13]	Case of primary mediastinal germ cell tumor, successfully treated with chemotherapy and curative resection	Primary mediastinal germ cell tumor	Curative resection after three courses of combination chemotherapy (cis-platinum, etoposide, bleomycin and adriamycin).	6 months after surgery
**Watanabe et al,** [14]	A case of mediastinal non seminomatous germ cell tumor successfully treated with chemotherapy and curative surgery	Mediastinal non seminomatous germ cell tumor	Surgery three courses of chemotherapy with CDDP, Bleomycin and etoposide	10 months after surgery
**Shiina et al,** [15]	Mediastinal non seminomatous germ cell tumor successfully treated with chemotherapy and curative surgery; report of a case	Mediastinal non seminomatous germ cell tumor	Surgery After 3 courses of chemotherapy with cisplatin and etoposide	2 years

## Conclusion

Primary mediastinal non seminomatous germ cell tumors is a clinical and biologic entity that should be distinguished from other germ cell tumors. About 40% of these patients can envisage long-term survival with modern therapy that includes cisplatin-based chemotherapy followed by surgical resection of residual masses. Predictive factors and improvement in therapy are required for these patients.

## Consent

Written informed consent was obtained from the patient for publication of this Case Report and any accompanying images. A copy of the written consent is available for review by the Editor-in-Chief of this journal.

## Abbreviations

BEP: Bleomycin, etoposide and cisplatin
IGCCCG: International germ cell cancer collaborative group
G-CSF: Granulocyte colony-stimulating factor.
